# The relationship between happiness and quality of life: A model for Spanish society

**DOI:** 10.1371/journal.pone.0259528

**Published:** 2021-11-03

**Authors:** Víctor-Raúl López-Ruiz, Nuria Huete-Alcocer, José-Luis Alfaro-Navarro, Domingo Nevado-Peña

**Affiliations:** 1 Department of Spanish and International Economics, Econometrics and History and Economic Institutions, University of Castilla-La Mancha, Albacete, Spain; 2 Department of Political Economy and Public Finance, Economic and Business Statistics and Economic Policy, University of Castilla-La Mancha, Albacete, Spain; 3 Department Business Administration, University of Castilla-La Mancha, Ciudad Real, Spain; Rzeszow University of Technology: Politechnika Rzeszowska im Ignacego Lukasiewicza, POLAND

## Abstract

A key goal for society as a whole is the pursuit of well-being, which leads to the happiness of its individual members; as such, it is of critical socioeconomic relevance. In this regard, it is important to study which factors primarily affect the happiness of the population. In principle, these factors are associated with income level and residential and job stability, or more specifically, citizens’ quality of life. This research, which is based on a multidimensional concept of quality of life, uses a regression model to explain the dependence of Spaniards’ happiness on the well-being or quality of life provided by their work, their family situation, their income level and aspects of their place of residence, among other factors. The data were collected through an anonymous survey administered to a representative sample of Spanish citizens. The methodology used approaches the intangible concept of happiness as resulting from different individual and social causes selected from dimensions addressed in the literature, and calculates their effects or importance through regression coefficients. One of the findings is that people with the highest level of well-being or quality of life in the most important dimensions mostly claim to be happy. With respect to gender, it has a significant influence on the dimensions included in the model of citizen happiness and on personal issues. It is also shown that the outbreak of the Covid-19 pandemic negatively influenced the quality of life of Spanish citizens and therefore their happiness.

## Introduction

Citizen’s quality of life, in an urban and community context, has become a central element of politics in most countries of the European Union [1]—so much so that it is the subject of extensive debate in different scientific fields. In sociology, quality of life is interpreted as the subjective understanding of well-being taking into account individual needs and perspectives; in economics, it is standard of living; and in medicine, it is the relationship between health and disease, along with the factors that have an impact on a healthy lifestyle. The health factor in quality of life is often elevated above other elements, although the concept of quality of life needs to be understood more broadly [2].

Life satisfaction is a subjective assessment of quality of life in general and is an indicator of subjective well-being [3, 4], which is seen as synonymous with happiness when it refers to how people feel and think about their lives [4, 5]. The topics of life satisfaction and happiness are currently attracting a good deal of attention from researchers in social sciences, psychology, philosophy and economics [6]. Most researchers use the word happiness carefully to convey its particular meaning: being happy is not just about being cheerful; it is a special feeling that is precious and extremely desirable, but difficult to attain [2]. Much of the research to date has focused on establishing objective methods for analysing quality of life and well-being, relying on geographical and socioeconomic aspects related to quality of life, well-being and happiness, with a particular emphasis on the impact of social and spatial inequalities, and social justice [7].

At the same time, however, there are many studies that consider more subjective aspects by means of social surveys [8], with citizens rating their well-being, health, life satisfaction and happiness in general [9]. Happiness is part of lived experience and everyday life [10]. However, happiness is also related to other socioeconomic aspects, such as individuals’ job satisfaction, which can have important implications for both individuals and organizations [10, 11]. There is evidence that life satisfaction, and therefore happiness, depends on the type of job a person has [12]. Furthermore, we should take into account the characteristics of urban areas, as they are centres of economic activity and consumption, where a high quality of life can attract human capital and develop aspects that bring about economic growth and foster well-being [13]. In this vein, [14] argue that, in order to achieve said growth and ensure people’s subjective well-being, the environmental quality of cities must be improved. In short, happiness directly depends on the different dimensions in which our (multidimensional) quality of life can be observed, fundamentally those relating to workplace and residential environments.

Based on this interpretation, the present study applies a subjective approach to analyse the influence on happiness simultaneously exerted by individuals’ internal features and external factors [15]. The aim of this study is to gain a better understanding of the influence of certain determinants on the happiness of Spanish citizens, since individuals tend to actively select their place of residence in light of the job opportunities, public goods and services they provide [16]. Thus, the choice of where to live is associated with an individual’s social and economic prospects [17] and therefore with his/her pursuit of happiness [9]. This is what determines the success or failure of cities or municipalities in providing opportunities for residents to attain a comfortable quality of life, with the goal being not only to attract new residents but also to encourage existing ones to stay. This situation calls for certain actions to ensure inhabitants’ satisfaction with life in the city where they live [1]. Furthermore, working conditions and even the income gained through work are seen as clear assets in a possible model of happiness [14, 18, 19]. Happiness at work, which covers workplace relations and the individual’s self-esteem and assessment of their job, leads to life satisfaction and therefore happiness. But a comprehensive model of happiness must account for the personal issues related to each individual, considered fundamental by [20], such as personal development, physical and mental conditions, and even spirituality.

In summary, we present a study of Spaniards’ preferences regarding different dimensions of quality of life for achieving happiness, and we also assess differences by gender. Using a survey of this population and a representative sample, we determine the main, significant relationships with the different dimensions, primarily relating to work and the place of residence. To do so, we run a regression model in which happiness is explained by these external or social variables, while the influence of individual’s internal factors is approximated from the error. Due to the time frame of the analysis, we are also able to evaluate the possible effect of the pandemic on quality of life and thus individuals’ happiness. A negative impact will doubtlessly be observed, due to the adverse situation in health, social capital and/or economic factors [21]. We use a model of happiness in which residential safety, individuals’ family, work and financial situation, their immediate surroundings, care for the environment and the culture and sports on offer all play an important role. The study proposes a conceptualization of the model for measuring happiness; an applied estimation method and the results, analysing differences by gender; and the conclusions drawn. We also examine the relevance of the issues inherent to the individual in this search for happiness, which represents a novel methodological approach.

### Background on happiness and development of hypotheses

Since the 1960s, the analysis of people’s quality of life has attracted the attention of researchers from many disciplines. Specifically, in the last decade there has been growing academic interest in quality of life, which in turn has included a series of studies that investigate well-being and happiness [22], with the latter becoming an important indicator of a country’s development.

It is generally believed that improving national happiness is the ultimate purpose of economic development [23]. In our case, individual happiness or the quality of life of a society is a key indicator of growth, and is broader and more complex than the aggregate measure of production. Thus, the measurement and analysis of happiness are becoming increasingly important in the social sciences [24], where there have been numerous attempts to define, measure and analyse subjective measures of happiness from the perspective of different academic disciplines, ranging from neuroscience and psychology to philosophy and economics [25]. For example [26, 27], hold that happiness reveals the individual’s assessment of the general aspects of his/her life and situation, and how much an individual likes the life he/she lives. In this context, the central concept of happiness is the subjective assessment of one’s life, or life satisfaction [28]; hence [29], believe that happiness can be measured through “Satisfaction with life in this city”. This way of measuring happiness finds support in the studies of [30–32]. In addition [33], view happiness as the experience of satisfaction, and this satisfaction can come from everything around a person. Accordingly, the place of residence affects happiness [34].

The literature review conducted for this research reveals the intermingling of the terms happiness and quality of life, as they are very closely linked; the same happens with happiness and well-being, in the subjective sense, which are considered equivalent concepts [35]. Nevertheless, there are some studies that make a clear distinction between quality of life and happiness, using elements such as income to measure quality of life [36]; others use employment [18, 37]; or the residential environment, physical and mental health, education, recreation and leisure, crime or security, and social belonging [38, 39]. In other studies, quality of life is seen as related to more abstract issues such as freedom, human rights and happiness [40], which makes it difficult to differentiate between quality of life and happiness. But equally we are seeing the emergence of many studies that use social surveys to examine more subjective aspects [8], with citizens rating their well-being, health, life satisfaction and happiness in general [9]. Thus, it has been shown that happiness is one of the key factors in subjective well-being and overall life satisfaction [5, 28, 41–43], as it is interwoven with and embedded in the cultural context where the individual lives [44]. As such, the current view of urban, economic and social policy on cities is becoming increasingly important [1] in determining the happiness of their residents [16, 39]. Indeed, people’s place of residence affects every aspect of their day-to-day life and therefore their happiness [16, 45, 46].

In this study, we adopt a subjective approach, based on the idea that Spaniards’ happiness—measured through their response to the statement “I feel satisfied in my place of residence”—depends on different types of factors. These are mostly drawn from the model of the different dimensions of quality of life which [47] refer to as important areas of life. To that end, we apply the quality of life model proposed by [48], which takes a subjective, general, multidimensional approach, with some of the characteristics relating to quality of life analysed for Spanish society (Table 1).

**Table 1:** Quality of life dimensions.

Factor	Dimensions/Variables
**Life Satisfaction, trust and safety**	Family and standard of family life (situation).
	Residence, accommodation
	Job, employment situation
	Safett and confidence in the environment and city
	Pollution, cleaning
**Mobility, culture and sports**	Accessibility and public transport
	Culture and spaces for development
	Sports and spaces for leisure and sports practices
**Integration and social sustainability**	Integration of foreigner
	Environmental commitment
	Accessibility to housing
**Public service**	Evaluation of the welfare state
	Administration efficiency

Source: Adapted from [48].

The influence of the different quality of life factors on a citizen’s happiness calls for a multidimensional approach that allows us to include a set of potential factors. We explore these in depth below, proposing the hypotheses to be tested in this study.

### Happiness and the family situation

[1] demonstrates that household composition and the length of time a family remain living in a city are not associated with satisfaction with life in that place. However, other studies [49] report that life satisfaction is significantly correlated, albeit weakly, with the family situation, essentially with the composition of the family (parents’ marital status, number of children, etc.). For example, the size of the family unit has a positive impact on individual happiness [4]. [50] state that only when families have their first child is a positive effect on their happiness observed. In this vein, researchers such as [51] indicate that having children is negatively related to subjective well-being, due to the negative impact on financial satisfaction.

Generally speaking, family-related aspects have an influence on happiness, as do demographic factors at the individual level: marital status, education, unemployment, disability, age and sex [14]. The happiness of older people is more vulnerable when they live alone than when they live with family [18]. Therefore, we propose the first hypothesis (H1) that a favourable family situation, in which family members are united and support one other, will have a positive effect on the happiness of each individual family member.

### Happiness and trust in one’s place of residence

People normally choose to live where they can feel happy with what they do in their daily life, such as their job, have confidence in their surroundings, and enjoy the services on offer that are accessible from their place of residence, such as healthcare and education. In addition to offering competitive opportunities to achieve a better financial situation and thus higher standard of living, the place where a person chooses to live can also influence a person’s happiness and well-being [46]. The migration flows from rural to urban areas in the last century were primarily motivated by this issue, and it is reflected in the inhabitants of large cities and in cities’ urban planning. In this vein, studies such as that by [52] claim that urban green spaces in cities help to assure citizens’ happiness by enhancing their physical and mental health. In recent years, changes have been made to global policy with efforts to build more urban green spaces, aimed at creating comfortable living environments and thereby improving quality of life in cities. This has been shown by numerous studies [53, 54], which find greater increases in well-being and therefore happiness in cities due to the job opportunities, public goods and services they provide [16].

This issue, along with the type of urbanization of a city, including streets and buildings, has proven to be equally important for predicting happiness. Thus, we are seeing a shift in subjective well-being with the development of the economy in large cities compared to smaller, rural areas [54]. Conversely, other studies, such as that by [46] demonstrate that urban development, in the sense of the choice between rural or urban places of residence, does not directly affect happiness. As such, we propose a second hypothesis (H2), which holds that one’s place of residence (rural or urban), and trust in those surroundings, urban planning and local residents—that is, one’s immediate socio-residential circle—has a direct and positive effect on one’s happiness.

### Happiness and the employment situation

Another key factor that exerts an influence on happiness is a person’s employment situation [16, 55]. Job satisfaction, broadly referring to the degree to which people like their job [56] also forms part of this issue. However, little is known about the relationship between happiness and how happy people are with their job [11]. [57] report that employees’ orientation to happiness is significant for achieving well-being or happiness at work.

Furthermore, there are studies that differentiate between happiness in one’s personal and professional life. When employees lack support in doing their job, it increases their unhappiness and they end up in a frustrating situation [10]. But not having a job is also considered a driver of unhappiness, with the unemployed being far less happy than employed people [4, 58].

Another aspect to take into account in workers’ happiness is the type of job they do [12] identify differences in the association between orientations to happiness and life satisfaction across occupation types.

We thus propose the third hypothesis (H3) which posits that working and favourable workplace conditions have a positive and significant effect on an individual’s happiness.

### Happiness and the financial situation

Over the past two decades, there has been a marked rise in economic studies of happiness, particularly those related to the effect of income on happiness [7, 8, 16, 19, 38, 55, 59–61]. In economics, happiness is defined as a benefit: a rise in income can increase people’s utility levels, leading to a higher level of happiness [4, 23].

Numerous studies have shown that most people with higher income levels have higher subjective well-being, although their happiness increases to a lesser extent [58]. However, other studies indicate that the impact of the income gap on happiness is unclear [23]. It could be the case that people with lower incomes have greater future prospects, which would encourage them to work much harder to improve their happiness [62].

On the other hand, if we focus on the place of residence, distinguishing between urban and rural, studies such as that by [23] found that the difference in income led to a significant decrease in residents’ subjective well-being. However, income inequality has a greater influence on the happiness of urban residents than on that of residents in rural areas [23]. As such, a household’s financial situation and the type of community are significantly correlated [1].

Therefore, the fourth hypothesis (H4) that we propose to examine is whether the financial situation has a significant and positive relationship with happiness, through the effect of subjective social well-being.

### Happiness and safety

The socioeconomic and cultural conditions and prospects of an individual’s city and neighbourhood of residence are important [1]. Inhabitants socioeconomic characteristics play an important role in satisfaction with the neighbourhood, pointing to the critical relevance of policies aimed at strengthening and sustaining local communities [3].

When citizens assess neighbourhood-related problems, they tend to significantly associate them with their satisfaction with living in that particular area, although such problems are not significant when it comes to their assessment of the city [1]. However, other studies have found that a positive attitude towards other citizens is positively correlated with the satisfaction of those who live in the city in question [63], and a positive social attitude towards neighbours is positively related to satisfaction with the neighbourhood [64] and with the local area [65]. Moreover, other studies have found that households with children differ from childless households in their perception of satisfaction with the neighbourhood or local area [65].

According to [64], a higher income level is associated with higher neighbourhood satisfaction; however, they find no relationship between housing satisfaction and neighbourhood satisfaction. On the other hand, a positive relationship has been found between subjective well-being and home ownership [66].

In this context, the fifth hypothesis (H5) addresses the existence of a positive and significant relationship between perceived safety in one’s place of residence and the individual’s happiness.

### Environment, climate change and happiness

There is limited evidence that momentary happiness is associated with immediate urban environments. [67] demonstrate that momentary happiness is influenced by immediate microenvironment variables and built environment characteristics, including temperature and noise. Similarly, the relationships between well-being and environmental factors are prompting a growing interest in the fields of psychology, health, conservation and economics [67, 68]. However, the lack of attention paid to the city environment points to a need for research to understand how different aspects of the environment impact happiness over a lifetime [69]. In the same vein, there are reasons to believe that the natural environment is positively related to well-being, health and happiness. Natural environments can increase happiness by facilitating and encouraging—for practical, cultural and/or psychological reasons—behaviour that is physically and mentally beneficial, including physical exercise, recreation, and social interaction; conversely, knowledge of a local environmental problem and its negative effects on human health and the ecosystem could directly reduce levels of happiness [68].

Thus, citizens’ perceptions of air pollution can influence their happiness [70]. [68] provide evidence that citizens are significantly happier outdoors, in any type of green or natural habitat, than in an urban environment. Indeed, some studies have explored how the dissemination of information on air quality in cities positively affects the happiness of citizens and their economic development [14].

Therefore, we propose a sixth hypothesis (H6) on sustainability, the environment, pollution and the happiness of the individual, positing a positive and significant relationship between happiness and an improvement in the environment and sustainable green policies.

### Other factors relating to accessibility, leisure and well-being (public services)

The benefits of leisure experiences (including social, physical, personal and psychological benefits) are among the main factors affecting quality of life [71]. According to [13], urban spaces are no longer centred around improving the infrastructure and transport connectivity of large cities; rather, there is a growing focus on other aspects such as economic competitiveness, culture and environmental values. In this regard, studies such as that of [69] analyze whether the provision of services within cities contributes to the happiness of their residents. Their results show that cities should focus on providing quality services, including good surveillance, schools, access to health services, easy access to transport services and cultural and sporting opportunities. The provision of such services underpins the success or failure of cities to provide opportunities for residents to secure a comfortable quality of life. Thus, there is a close relationship between the progress that is being made in the standard of living and the urbanization of the place of residence. Accordingly, some scholars have concluded that we are happier in cities [53], as people tend to choose their place of residence according to the job opportunities, goods and public services they provide [16].

In this regard, the seventh hypothesis (H7) that we propose is a significant and positive relationship between the citizen’s happiness and the range and accessibility of cultural and sports services on offer.

### The Covid-19 pandemic and happiness

Happiness and life satisfaction are determined not only by personal aspects and life events, but also by circumstances external to the individual occurring at a certain point in time. In this regard, it can be stated that the Covid-19 pandemic is having a negative impact on multiple aspects of life for people around the world. However, it has been found that asking about the Covid-19 pandemic in surveys leads to positive changes in both momentary happiness and overall happiness with life [72].

There is a growing number of studies examining the effect of Covid-19 [21]. The results of some studies indicate that lockdowns have a significant and negative impact on happiness [21]. However, there are others that report that attitudes towards the Covid-19 pandemic in terms of the credibility of real-time data updates and society’s confidence in the handling of the pandemic are associated with lower levels of depression and higher levels of happiness [6].

Due to the impact that the pandemic is having on society, including people being confined to their homes, rural areas are offering new possibilities as places to live; indeed, it has been shown that such places are safer than urban localities in times of pandemic [73]. This represents an opportunity to address the issue of “Empty Spain", as the possibility of working from home can provide a boost to depopulated rural municipalities [74]. Indeed, a good many jobs can feasibly be done from home and thus from many of these rural areas [75].

We therefore propose the eighth hypothesis of this research (H8), which posits the existence of a negative and significant relationship between the effects of the pandemic and the happiness of individuals.

All the variables presented above are aimed at assessing quality of life and its influence on the happiness of the individual (Spanish citizen). Of the proposed hypotheses, the first seven posit a positive and significant relationship between the analysed variables and the happiness of the individual. The last hypothesis, relating to the pandemic, posits a negative and significant effect.

Furthermore, there is another set of conditions or variables, which we can identify as personal and inherent to the individual, relating to spirituality and physical and mental health [76]; these factors shape people’s development and personal growth, their sense of the meaning of life, self-respect and self-esteem, and as such are expected to have a significant influence on their happiness. In this regard, authors such as [77] develop a theoretical model of quality of life that distinguishes conditions of physical well-being, health and self-esteem within the category of the individual’s internal environment of quality of life. Although such issues are difficult to measure, even by means of an anonymous questionnaire, by running a regression model we can isolate the effects through the error or the variables not explicitly included in the model. By doing so, we can test whether this personal dimension influences the relationship with happiness. Below we explain the proposed method, the specification of the model and the measurement of all of the social and personal effects that allow us to test whether the hypotheses are supported.

## Materials and methods

To test the hypotheses on the relationships between quality of life factors and individual happiness, and by extension social happiness, we establish a model for working-age Spanish citizens over 16 years old, with a sample generated through a questionnaire. The model includes the quality of life dimensions selected on the basis of their theoretical relevance and the statistical significance registered by their partial correlation and regression coefficients. Specifically, the equation is specified for each individual surveyed, with the quality of life dimensions that influence their happiness.

The data were collected between 2nd July and 8th September 2020 using an anonymous online questionnaire distributed through mailing lists and social networks. The final sample obtained was composed of 933 responses from across Spain. It should be borne in mind that this period coincides with the tail end of the first wave of the pandemic, which had lasted over three months, and the return to a “new normal” with the 14-day notification rate of new cases per 100 000 inhabitants well below 10 and minimum values for hospitalizations and deaths, according to information from the Ministry of Health. The tabulation method chosen was a 10-point Likert scale measuring citizens’ degree of satisfaction with the different aspects of quality of life and well-being in their places of residence and workplaces (with 1 being “not at all satisfied” and 10 being "very satisfied"), relating to the dimensions of the proposed model of quality of life and happiness. Questions related to Covid-19 and its effect on quality of life were also included.

Once the information had been collected, classified and tabulated, we ran an Ordinary Least Squares (OLS) regression using EViews 11 software. This type of estimation has been carried out for various quality of life factors, aiming to explain a high proportion of the variance in the dependent variable [47].

This method can be used to quantify for a set of individuals the dependence between the dependent variable “happiness” (Hp) and the explanatory or independent variables identified in the literature review and presented in the survey. We chose between them on the basis of a descriptive statistical analysis (Table 2 shows the partial correlation coefficients: family situation (Fm), trust in one’s close circle and neighbours (Tr), environmental protection policies (En), provision of culture and sports and spaces for the population to engage in these activities (CS), the safety provided by the place of residence (Sf), financial situation (In), employment situation, along with the individual’s assessment of doing the job, known in the literature as “happiness at work” (Hw), and the effects of the Covid-19 pandemic (C19). Therefore, to test the proposed relationships all together the following regression model is constructed:

Hpi=β0+β1·Fmi+β2·Tri+β3·Eni+β4·CSi+β5·Sf+β6·In+β7·Hwi+β8·C19i+ui

**Fig 1:**
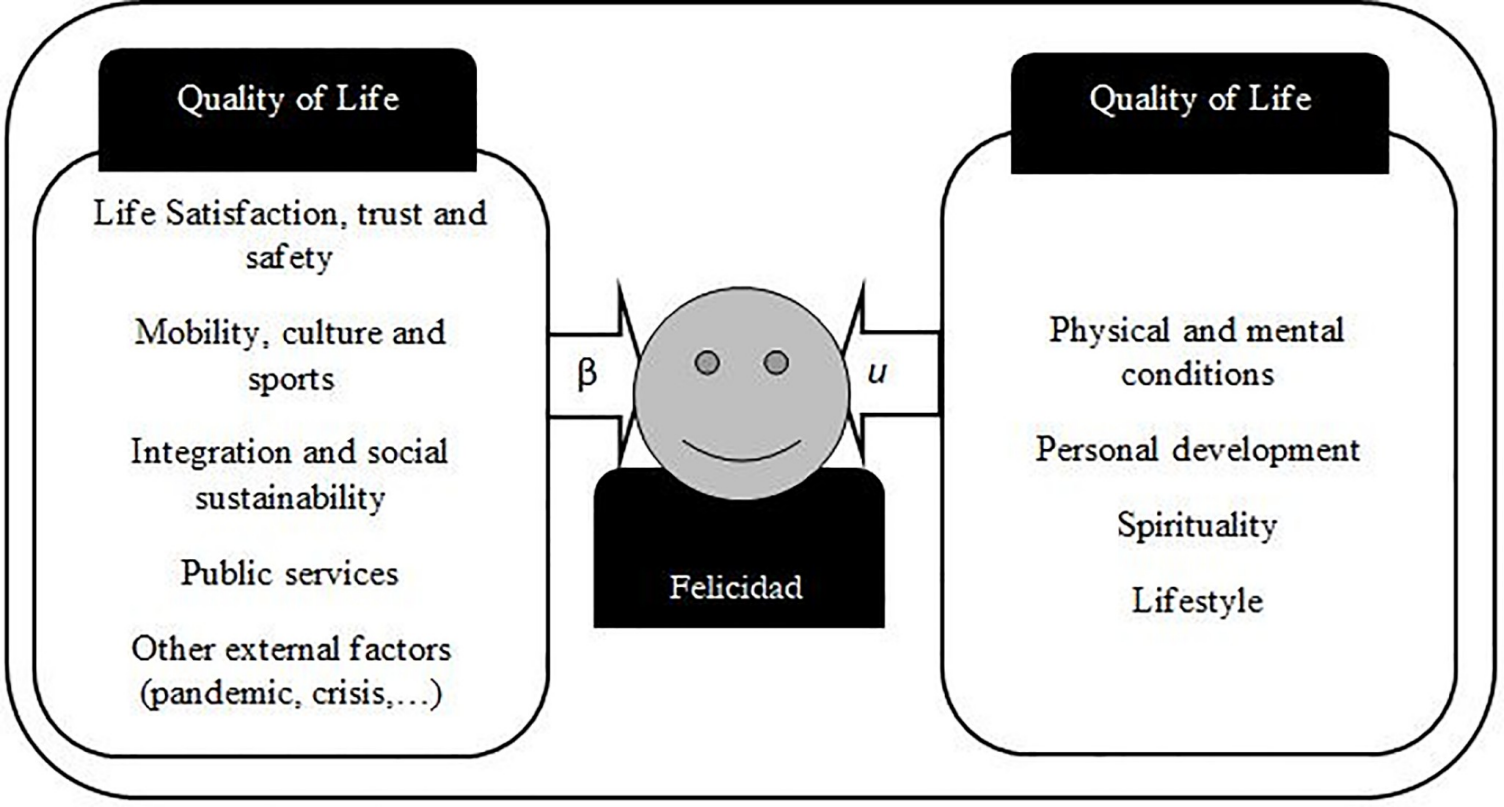
Happiness model structure.

Where the dependent variable is the happiness of individual i (Hpi). The β coefficients indicate the relevance of each independent variable with respect to the happiness of the individual, with the constant term being an autonomous factor of happiness justified by the individual minimum. The relationship is linear and includes a random variable *u* that captures, in line with our happiness model, the variables referring to the individual’s personal situation that are not incorporated in the quality of life dimensions but that together may be relevant (Fig 1). However, regarding the behaviour of this random variable, it is normally distributed with zero expectation value, are uncorrelated, and with constant variance.

**Table 2:** Descriptive analysis.

N	Hp	*Fm*	*Tr*	*En*	*CS*	*Sf*	*In*	*Wh*	*-C19*	
**i 933**	**8.118**	**8.154**	**7.220**	**7.094**	**7.302**	**8.037**	**7.197**	**7.332**	**6.889**	**Av**
	*1*.*725*	*1*.*796*	*1*.*874*	*2*.*075*	*1*.*894*	*1*.*540*	*2*.*170*	*2*.*240*	*2*.*351*	***σ***
	*21*.*25*	*22*.*03*	*25*.*95*	*29*.*25*	*25*.*93*	*19*.*16*	*30*.*14*	*30*.*53*	*34*.*13*	***c*.*v*.**
**Male 359**	**8.045**	**8.134**	**7.167**	**7.000**	**7.276**	**7.989**	**7.387**	**7.591**	**6.869**	**Av**
	*1*.*764*	*1*.*709*	*1*.*938*	*2*.*064*	*1*.*967*	*1*.*692*	*2*.*045*	*2*.*067*	*2*.*391*	***σ***
	*21*.*92*	*21*.*02*	*27*.*04*	*29*.*49*	*26*.*80*	*21*.*18*	*27*.*68*	*27*.*24*	*34*.*81*	***c*.*v*.**
**Female 574**	**8.164**	**8.167**	**7.252**	**7.153**	**7.319**	**8.068**	**7.078**	**7.172**	**6.901**	**Av**
	*1*.*699*	*1*.*847*	*1*.*832*	*2*.*080*	*1*.*846*	*1*.*435*	*2*.*236*	*2*.*325*	*2*.*326*	***σ***
	*20*.*81*	*22*.*63*	*25*.*26*	*29*.*07*	*25*.*23*	*17*.*79*	*31*.*59*	*32*.*42*	*33*.*71*	***c*.*v*.**

The model of citizen happiness therefore incorporates the key factors of citizen quality of life that influence life satisfaction, with the importance of the personal factors determined through the error, or the proportion of variance not explained. That is, the model explicitly analyses the factors related to social behaviour in the residential and labour spheres, including those relating to external circumstances, while personal issues account for the part of the happiness that is inherent to the individual and that cannot be directly quantified but can be measured through the proportion of the variance that is not explained by the model.

## Results and discussion

First, Table 2 shows the main descriptive statistics of the variables that have the most significant relationships with the variable Hp for the whole sample, and broken down by gender.

The descriptive analysis of the dependent variable shows that, although there is a gender gap in favour of women, the difference is not significant. However, in the set of independent variables, there are clear differences in favour of men in the work-related issues of income and the assessment of their job, stemming from the gender inequality in the Spanish labour market. Although less significant, in the issues regarding personal relationships with one’s surroundings it is women who register higher average values (av). Regarding the dispersion, measured through the standard deviation (σ) and the coefficient of variation (CV), it is similar for all items, but greater for work-related issues, income and pandemic effects, while the lowest values correspond to the relationship between safety and life satisfaction. The coefficients of variation, means and standard errors for the selected variables can be seen in Table 2.

By estimating the regression model in Equation 1, which yields the results summarized in Table 3, we can determine the happiness model for Spanish society in terms of priorities for quality of life. It seems clear that in this model, gender is a determinant of priorities regarding happiness. For men, 55.2% of the variance is explained by these factors, while the corresponding value for women is only 41.5% (values of the coefficient of determination R^2^).

**Table 3:** Estimated relationship among happiness: General and by gender.

Hp	*C*	*Fm*	*Tr*	*En*	*CS*	*Sf*	*In*	*Hw*	*C19*	R^2^
**Total**	0.8805	0.2859	0.1055	0.1319	0.1412	0.1619	0.0693	0.0546	-0.003	**0.4496**
**i 933**	(2.94)	(10.52)	(3.90)	(5.52)	(5.74)	(4.69)	(2.73)	(2.29)	*(-0*.*18)*	
**r**_**Hp,X**_		0.5012	0.4412	0.3876	0.3925	0.4474	0.4158	0.3763	0.0077	
**Test normality residuals Jarque-Bera 124.89, p-value: 0.0000**										
**Male**	0.5929	0.1659	0.1696	0.1690	0.1273	0.0580	0.0981	0.1969	0.0150	**0.5517**
**i 359**	*(1*.*38)*	(3.74)	(4.19)	(4.73)	(3.36)	*(1*.*18)*	(2.15)	(4.53)	*(0*.*56)*	
**r**_**Hp,X**_		0.4578	0.5421	0.4528	0.4555	0.4881	0.5427	0.5777	0.056	
**Test normality residuals Jarque-Bera 33.15, p-value: 0.0000**										
**Female**	1.2700	0.3426	0.0570	0.1015	0.1334	0.2083	0.0585	0.0039	-0.206	**0.4154**
**i 574**	(3.11)	(10.03)	*(1*.*61)*	(3.22)	(4.18)	(4.36)	*(1*.*91)*	*(0*.*14)*	*(-0*.*87)*	
**r**_**Hp,X**_		0.5284	0.3713	0.3445	0.3488	0.4170	0.3475	0.2693	-0.025	
**Test normality residuals Jarque-Bera 68.03, p-value: 0.0000**										

Note: t-statistic values in parentheses, in italics, are not significant at the 95% confidence level.

As a contribution to the scientific understanding of happiness, the following general observations can be made for Spanish citizens: all of the independent variables relating to life satisfaction are significant, as shown by the individual significance values (t-values), and have a direct relationship with happiness. We can thus accept the first seven hypotheses proposed, regarding the family situation (Fm), trust (Tr), the environment (En), culture and sport (CS), safety (Sf), financial situation (In), and job satisfaction, job assessment and working conditions (Hw). The pandemic is not found to be significant, although it has a negative sign. Therefore, H8 is not supported. This may be due to the time when the survey was carried out, despite the fact that the average value for this element in terms of quality of life was 6.9 out of 10. Lastly, regarding the personal issues not analysed in the model, the value of the coefficient of determination indicates that they account for approximately half the effect on the happiness of the Spanish individual in 2020: the R^2^ is 0.45 and the residuals meet the requirement of normality (Jarque-Bera test: 124.89, p-value: 0.0000).

Looking closely at the effects, we can see that the most influential variable is the one referring to individuals’ closest circle, the family (Fm). It registers the largest significant effect (t-value 10.5) for both genders, although in this case the β effect is clearly higher in women (0.34 to 0.16 for women and men respectively).

Similarly, the items relating to the environment and sustainability (En) are relevant in all cases, but the effect is somewhat higher for men.

The safety of one’s surroundings, of one’s city, town or neighbourhood (Sf), is another clearly significant variable, but not for the male gender. The situation is reversed when it comes to trust in people, in terms of personal relationships (Tr): in this case, the effect is significant, but if we break it down by gender, it is men for whom the effect is significant, as shown by the t-value. The issues of trust and safety are thus relevant and are one reason why, if we extract the information by the size of the place of residence, the results point to small cities or large towns as being advantageous for attaining a higher quality of life and greater life satisfaction.

Finally, in terms of items regarding the individuals’ surroundings, we see a uniformly significant effect of the culture and sports variable (CS) in all equations.

The variables reflecting the workplace and financial situation merit particular mention, as there is a clear gender gap relating to the labour market. Both the income variable (In) and satisfaction with one’s job and workplace relations (Hw) are favourable for the male gender. For women, they are not significant (t value 0.19).

In summary, in terms of fit, more of the variance of the dependent variable is explained for men (R^2^ 0.55). The factors driving this relate to both the residential and the workplace environment. In the latter case, the assessment of their job and status at work clearly bring men happiness. However, the female gender is more influenced by the family situation and the safety of their place of residence, although these explain a smaller proportion of the variance (R^2^ 0.415). As such, individual factors not explicitly incorporated in the model, such as physical, mental and personal development issues, are more important for women’s happiness.

## Conclusions

The study of happiness as a variable that is dependent on certain social factors has become critical, even more so in precarious situations such as the one generated by the current Covid-19 pandemic. In this regard, the literature reviewed confirms that the most extensively analysed factors in this relationship are those relating to financial and labour spheres, and to a lesser extent aspects of one’s place of residence.

The model of happiness proposed here is theoretically grounded and empirically tested through regression analysis, and represents a novel contribution to the literature in that it includes both explicit social factors or those relating to interpersonal relationships in one’s place of residence, surroundings and workplace, as well as implicit personal issues, inherent to the individual.

Based on data from a questionnaire, we have been able to quantify the main factors though which Spanish society attains happiness. The regression analysis provides evidence to support the hypotheses that posit the significance of factors such as one’s family situation, trust in neighbours, the safety of the place where one lives, culture, sport, sustainability, an unpolluted environment, and the one’s financial and labour situation. They all exert a positive influence on citizens’ happiness; however, the model reveals a divergence when analysing results by gender.

The gender gap in the Spanish labour market plays a decisive role in the factors relating to quality of life assessment in the happiness model. In particular, job assessment, workplace relations and happiness at work, along with one’s financial situation, are determining factors for men, but do not show the same effect for women.

On the other hand, the pandemic does not show a significant effect on life satisfaction, despite the fact that 80% respondents admitted to feeling affected in their personal, family or work sphere; this may be because the survey was conducted in the summer, when the first three-month wave of the pandemic was coming to an end.

This study opens up new lines of future research that will depend on the availability of information. An in-depth analysis is needed of the longer-term influence that the Covid-19 pandemic has had on these factors, in order to determine whether this situation has changed the key factors for citizen’s happiness, or even their preferences for cities with lower population density. To that end, a survey is currently being prepared to collect a new sample at a different time.

## References

[1] Węziak-Białowolska D., Quality of life in cities–Empirical evidence in comparative European perspective. Cities, 2016. 58: p. 87–96.

[2] A., S.D.a.J., The Concepts of Quality of Life and Happiness–Correlation and Differences. Engineering Economics, 2009. 63(4).

[3] Baum S., Arthurson K., and Rickson K., Happy People in Mixed-up Places: The Association between the Degree and Type of Local Socioeconomic Mix and Expressions of Neighbourhood Satisfaction. Urban Studies, 2009. 47(3): p. 467–485.

[4] Cuñado J. and de Gracia F.P., Environment and Happiness: New Evidence for Spain. Social Indicators Research, 2012. 112(3): p. 549–567.

[5] Diener E., Subjective well-being: The science of happiness and a proposal for a national index. American Psychologist, 2000. 55(1): p. 34–43. 11392863

[6] Lu H., Tong P., and Zhu R., Longitudinal evidence on social trust and happiness in China: Causal effects and mechanisms. Journal of Happiness Studies, 2020. 21(5): p. 1841–1858.

[7] Huang J., Income Inequality, Distributive Justice Beliefs, and Happiness in China: Evidence from a Nationwide Survey. Social Indicators Research, 2018. 142(1): p. 83–105.

[8] Simona-Moussa J., The Subjective Well-Being of Those Vulnerable to Poverty in Switzerland. Journal of Happiness Studies, 2019. 21(5): p. 1561–1580.

[9] Khan R.A. and Hussain S., Book Review: The Quality of Life and Policy Issues among the Middle East and North African Countries. Applied Research in Quality of Life, 2019. 15(3): p. 931–933.

[10] Singh S. and Aggarwal Y., Happiness at Work Scale: Construction and Psychometric Validation of a Measure Using Mixed Method Approach. Journal of Happiness Studies, 2017. 19(5): p. 1439–1463.

[11] Martínez-Martí M.L. and Ruch W., The Relationship Between Orientations to Happiness and Job Satisfaction One Year Later in a Representative Sample of Employees in Switzerland. Journal of Happiness Studies, 2016. 18(1): p. 1–15.

[12] Hofmann J., Gander F., and Ruch W., Exploring differences in well-being across occupation type and skill. Translational Issues in Psychological Science, 2018. 4(3): p. 290–303.

[13] Goerlich F.J. and Reig E., Quality of life ranking of Spanish cities: A non-compensatory approach. Cities, 2021. 109. doi: 10.1016/j.cities.2020.103013 33536696 PMC7809620

[14] Wang J., et al., Does mandatory air quality information disclosure raise happiness? Evidence from China. Energy Economics, 2021. 94.

[15] Yin C., et al., Happiness in urbanizing China: The role of commuting and multi-scale built environment across urban regions. Transportation Research Part D: Transport and Environment, 2019. 74: p. 306–317.

[16] Florida R., Mellander C., and Rentfrow P.J., The Happiness of Cities. Regional Studies, 2013. 47(4): p. 613–627.

[17] Baum S., Arthurson K., and Rickson K., Happy People in Mixed-up Places: The Association between the Degree and Type of Local Socioeconomic Mix and Expressions of Neighbourhood Satisfaction. Urban Studies, 2010. 47(3): p. 467–485%U http://journals.sagepub.com/doi/10.1177/0042098009351941.

[18] Hwang E.J. and Sim I.O., Association of living arrangements with happiness attributes among older adults. BMC Geriatr, 2021. 21(1): p. 100. doi: 10.1186/s12877-021-02017-z 33541268 PMC7860621

[19] Frey, B.S., What future happiness research?, ed. I.A.M.G.t.t.E.o. Happiness. 2021. 17–27.

[20] Maslow A.H., Toward a Psychology of Being. New York, Cincinnati. 1962, Toronto: Van Nostrand Reinhold.

[21] Greyling T., Rossouw S., and Adhikari T., The good, the bad and the ugly of lockdowns during Covid-19. PLoS One, 2021. 16(1): p. e0245546. doi: 10.1371/journal.pone.0245546 33481848 PMC7822257

[22] Li C.-L., Quality of life: The perspective of urban park recreation in three Asian cities. Journal of Outdoor Recreation and Tourism, 2020. 29: p. 100260.

[23] Li Z. and Xie Z., The Impact of Income Inequality and the Use of Information Media on Happiness. Open Journal of Social Sciences, 2020. 08(02): p. 128–142.

[24] Yang J., Liu K., and Zhang Y., Happiness Inequality in China. Journal of Happiness Studies, 2018. 20(8): p. 2747–2771.

[25] Ballas D., What makes a ‘happy city’? Cities, 2013. 32: p. S39–S50.

[26] Veenhoven R., Inequality Of Happiness in Nations. Journal of Happiness Studies, 2005. 6(4): p. 351–355.

[27] Diener E. and Seligman M.E.P., Beyond money: Toward an economy of well-being. Psychological science in the public interest, 2004. 5(1): p. 1–31. doi: 10.1111/j.0963-7214.2004.00501001.x 26158992

[28] Diener E.D., et al., The satisfaction with life scale. Journal of personality assessment, 1985. 49(1): p. 71–75. doi: 10.1207/s15327752jpa4901_13 16367493

[29] Arechavala N.S., Espina P.Z., and Trapero B.P., The economic crisis and its effects on the quality of life in the European Union. Social Indicators Research, 2015. 120(2): p. 323–343.

[30] Diener E., Inglehart R., and Tay L., Theory and validity of life satisfaction scales. Social Indicators Research, 2013. 112(3): p. 497–527.

[31] Diener E., The remarkable changes in the science of subjective well-being. Perspectives on Psychological Science, 2013. 8(6): p. 663–666. doi: 10.1177/1745691613507583 26173230

[32] Veenhoven R., Informed Pursuit of Happiness: What we should know, do know and can get to know. Journal of Happiness Studies, 2014. 16(4): p. 1035–1071.

[33] Moeinaddini M., et al., Proposing a new score to measure personal happiness by identifying the contributing factors. Measurement, 2020. 151.

[34] Cordero J.M., Salinas-Jiménez J., and Salinas-Jiménez M.M., Exploring factors affecting the level of happiness across countries: A conditional robust nonparametric frontier analysis. European Journal of Operational Research, 2017. 256(2): p. 663–672.

[35] Hirschauer N., Lehberger M., and Musshoff O., Happiness and Utility in Economic Thought—Or: What Can We Learn from Happiness Research for Public Policy Analysis and Public Policy Making? Social Indicators Research, 2014. 121(3): p. 647–674.

[36] Štreimikienė D. and Barakauskaitė-Jakubauskienė N., Sustainable development and quality of life in Lithuania compared to other countries. Technological and Economic Development of Economy, 2012. 18(4): p. 588–607.

[37] Iani L., et al., Happiness in Italy: translation, factorial structure and norming of the subjective happiness scale in a large community sample. Social Indicators Research, 2014. 118(3): p. 953–967.

[38] Zagorski K., et al., Does National Income Inequality Affect Individuals’ Quality of Life in Europe? Inequality, Happiness, Finances, and Health. Social Indicators Research, 2013. 117(3): p. 1089–1110.

[39] Zhang Y. and Wang P. The Relationship between the Degree of Urban Development and Human Happiness. in 2nd International Conference on Social Science, Public Health and Education (SSPHE 2018). 2019. Atlantis Press.

[40] Flynn P., Berry D., and Heintz T., Sustainability and quality of life indicators: Toward the integration of economic, social and environmental measures. The Journal of Social Health, 2002. 1(4): p. 274–286.

[41] Buss D.M., The evolution of happiness. American psychologist, 2000. 55(1): p. 15. 11392858

[42] Strack F., Argyle M., and Schwarz N., Subjective well-being: An interdisciplinary perspective. 1991.

[43] Moghnie L. and Kazarian S.S., Subjective Happiness of Lebanese College Youth in Lebanon: Factorial Structure and Invariance of the Arabic Subjective Happiness Scale. Social Indicators Research, 2011. 109(2): p. 203–210.

[44] Wang S.-Y., et al., What makes a meaningful life? Examining the effects of interpersonal harmony, dialectical coping, and nonattachment. Asian Journal of Social Psychology, 2018. 21(3): p. 198–204.

[45] Papachristou I.A. and Rosas-Casals M., Cities and quality of life. Quantitative modeling of the emergence of the happiness field in urban studies. Cities, 2019. 88: p. 191–208.

[46] Maricchiolo F., et al., The role of urbanization of place of living in the relation between individual features and happiness (El papel del desarrollo urbanístico del lugar de residencia en la relación entre las características individuales y la felicidad). PsyEcology, 2020. 11(2): p. 232–259.

[47] Bowling A. and Windsor J., Towards the good life: A population survey of dimensions of quality of life. Journal of Happiness Studies, 2001. 2(1): p. 55–82.

[48] Nevado-Peña D., López-Ruiz V.-R., and Alfaro-Navarro J.-L., Improving quality of life perception with ICT use and technological capacity in Europe. Technological Forecasting and Social Change, 2019. 148.

[49] Leto I.V., Petrenko E.N., and Slobodskaya H.R., Life Satisfaction in Russian Primary Schoolchildren: Links with Personality and Family Environment. Journal of Happiness Studies, 2018. 20(6): p. 1893–1912.

[50] Kohler H.P., Behrman J.R., and Skytthe A., Partner+ children = happiness? The effects of partnerships and fertility on well‐being. Population and development review, 2005. 31(3): p. 407–445.

[51] Beja E.L., Direct and indirect impacts of parenthood on happiness. International Review of Economics, 2015. 62(4): p. 307–318.

[52] Kwon O.-H., et al., Urban green space and happiness in developed countries. arXiv preprint arXiv:2101.00807, 2021. doi: 10.1140/epjds/s13688-021-00278-7 34094809 PMC8164893

[53] Okulicz-Kozaryn A. and Mazelis J.M., Urbanism and happiness: A test of Wirth’s theory of urban life. Urban Studies, 2018. 55(2): p. 349–364.

[54] De Neve J.-E. and Krekel C., Cities and happiness: a global ranking and analysis. World Happiness Report 2020, 2020: p. 14.

[55] Eren K.A. and Aşıcı A.A., The determinants of happiness in Turkey: Evidence from city-level data. Journal of Happiness Studies, 2017. 18(3): p. 647–669.

[56] Millán J.M., et al., Determinants of job satisfaction: a European comparison of self-employed and paid employees. Small Business Economics, 2011. 40(3): p. 651–670.

[57] Tandler N., Krauss A., and Proyer R.T., Authentic Happiness at Work: Self- and Peer-Rated Orientations to Happiness, Work Satisfaction, and Stress Coping. Front Psychol, 2020. 11: p. 1931. doi: 10.3389/fpsyg.2020.01931 32849134 PMC7426460

[58] Frey B.S. and Stutzer A., Happiness, economy and institutions. The Economic Journal, 2000. 110(466): p. 918–938.

[59] Dang Y., et al., How does growing city size affect residents’ happiness in urban China? A case study of the Bohai rim area. Habitat International, 2020. 97: p. 102120.

[60] Kollamparambil U., Happiness, Happiness Inequality and Income Dynamics in South Africa. Journal of Happiness Studies, 2019. 21(1): p. 201–222.

[61] Lim H.-E., et al., The Effects of Income on Happiness in East and South Asia: Societal Values Matter? Journal of Happiness Studies, 2019. 21(2): p. 391–415.

[62] Alesina A., Di Tella R., and MacCulloch R., Inequality and happiness: are Europeans and Americans different? Journal of Public Economics, 2004. 88(9–10): p. 2009–2042.

[63] Insch A., Managing residents’ satisfaction with city life: Application of Importance–Satisfaction analysis. Journal of Town & City Management, 2010. 1(2).

[64] Parkes A., Kearns A., and Atkinson R., What makes people dissatisfied with their neighbourhoods? Urban studies, 2002. 39(13): p. 2413–2438.

[65] Herting J.R. and Guest A.M., Components of satisfaction with local areas in the metropolis. The Sociological Quarterly, 1985. 26(1): p. 99–116.

[66] Zheng X., Yuan Z.-q., and Zhang X., Does happiness dwell in an owner-occupied house? Homeownership and subjective well-being in urban China. Cities, 2020. 96.

[67] Su L., et al., The impact of immediate urban environments on people’s momentary happiness. Urban Studies, 2021: p. 0042098020986499.

[68] MacKerron G. and Mourato S., Happiness is greater in natural environments. Global environmental change, 2013. 23(5): p. 992–1000.

[69] Wahl H.W., Iwarsson S., and Oswald F., Aging well and the environment: toward an integrative model and research agenda for the future. Gerontologist, 2012. 52(3): p. 306–16. doi: 10.1093/geront/gnr154 22419248

[70] Day R., Place and the experience of air quality. Health & Place, 2007. 13(1): p. 249–260. doi: 10.1016/j.healthplace.2006.01.002 16500135

[71] Park S., et al., Role of the Leisure Attributes of Shared Bicycles in Promoting Leisure Benefits and Quality of Life. Sustainability, 2021. 13(2). doi: 10.3390/su13020965 34123411 PMC8193797

[72] O’Donnell A., et al., Life satisfaction and happiness in patients shielding from the COVID-19 global pandemic: A randomised controlled study of the ’mood as information’ theory. PLoS One, 2020. 15(12): p. e0243278. doi: 10.1371/journal.pone.0243278 33306679 PMC7732102

[73] Morales A.M., et al., Covid-19.¿ Oportunidad para el mundo rural en España? Una reflexión. Actividad empresarial en la pandemia de la covid-19 N° 170, 2020: p. 167.

[74] Rodríguez J.D., Molina M.G., and Becerra L.A.C., Felicidad en la política pública: una revisión de literatura. Cuadernos de Economía, 2018. 37(73): p. 95–119.

[75] Anghel, B., EL TELETRABAJO EN ESPAÑA. p. 20.

[76] Lindström B., and Eriksson B. Quality of life among children in the Nordic countries. Quality of Life Research, 2(1), 23–32., 1993. doi: 10.1007/BF00642886 8490614

[77] Pukeliene V. and Starkauskiene V., Quality of Life: Factors Determining its Measurement Complexity. Engineering Economics, 2011. 22(2).

